# MicroRNA-33a regulates cholesterol synthesis and cholesterol efflux-related genes in osteoarthritic chondrocytes

**DOI:** 10.1186/s13075-015-0556-y

**Published:** 2015-03-05

**Authors:** Fotini Kostopoulou, Konstantinos N Malizos, Ioanna Papathanasiou, Aspasia Tsezou

**Affiliations:** Department of Cytogenetics and Molecular Genetics, School of Medicine, University of Thessaly, Biopolis, 41110 Larissa, Greece; Center for Research and Technology Hellas (CERTH), 6th Km Charilaou-Thermi Road, PO Box 60361, GR 57001 Thermi Thessaloniki, Greece; Department of Orthopaedics, School of Medicine, University of Thessaly, Biopolis, 41110 Larissa, Greece; Department of Biology, School of Medicine, University of Thessaly, Biopolis, 41110 Larissa, Greece

## Abstract

**Introduction:**

Several studies have shown that osteoarthritis (OA) is strongly associated with metabolism-related disorders, highlighting OA as the fifth component of the metabolic syndrome (MetS). On the basis of our previous findings on dysregulation of cholesterol homeostasis in OA, we were prompted to investigate whether microRNA-33a (miR-33a), one of the master regulators of cholesterol and fatty acid metabolism, plays a key role in OA pathogenesis.

**Methods:**

Articular cartilage samples were obtained from 14 patients with primary OA undergoing total knee replacement surgery. Normal cartilage was obtained from nine individuals undergoing fracture repair surgery. Bioinformatics analysis was used to identify miR-33a target genes. miR-33a and sterol regulatory element-binding protein 2 (*SREBP-2*) expression levels were investigated using real-time PCR, and their expression was also assessed after treatment with transforming growth factor-β1 (TGF-β1) in cultured chondrocytes. Akt phosphorylation after treatment with both TGF-β1 and miR-33a inhibitor or TGF-β1 and miR-33a mimic was assessed by Western blot analysis. Furthermore, we evaluated the effect of miR-33a mimic and miR-33a inhibitor on *Smad7*, a negative regulator of TGF-β signaling, on cholesterol efflux-related genes, ATP-binding cassette transporter A1 (ABCA1), apolipoprotein A1 (*ApoA1*) and liver X receptors (*LXRα* and *LXRβ*), as well as on matrix metalloproteinase-13 (*MMP-13*), using real-time PCR.

**Results:**

We found that the expression of miR-33a and its host gene *SREBP-2* was significantly elevated in OA chondrocytes compared with normal chondrocytes. Treatment of cultured chondrocytes with TGF-β1 resulted in increased expression of both miR-33a and *SREBP-2*, as well as in rapid induction of Akt phosphorylation, whereas TGF-β-induced Akt phosphorylation was enhanced by miR-33a and suppressed by inhibition of miR-33a, as a possible consequence of *Smad7* regulation by miR-33a. Moreover, treatment of normal chondrocytes with miR-33a resulted in significantly reduced *ABCA1* and *ApoA1* mRNA expression levels and significantly elevated *MMP-13* expression levels, promoting the OA phenotype, whereas miR-33a’s suppressive effect was reversed using its inhibitor.

**Conclusions:**

Our findings suggest, for the first time to our knowledge, that miR-33a regulates cholesterol synthesis through the TGF-β1/Akt/SREBP-2 pathway, as well as cholesterol efflux-related genes *ABCA1* and *ApoA1*, in OA chondrocytes, pointing to its identification as a novel target for ameliorating the OA phenotype.

## Introduction

Osteoarthritis (OA), the most common form of arthritis, is a chronic degenerative joint disease that affects millions of people worldwide [1]. It is thought of as a “joint failure” due to molecular changes that take place in all joint tissues [2]. OA is a complex disorder in which increased mechanical load and inflammation, combined with genetic predisposition, trauma and obesity, contribute to its initiation and progression [3].

OA is now considered as a disease with a variety of phenotypes, including the metabolic phenotype, because OA and the metabolic syndrome (MetS) are tied together in fundamental ways [4]. Their association is further supported by a number of studies linking OA to hypertension, type 2 diabetes and dyslipidemia, all characteristics of MetS [4,5], whereas common molecules seem to be involved in the pathophysiology of both OA and metabolic disturbances, highlighting OA as a new facet of MetS [6].

Imbalances of lipid traffic or metabolic homeostasis may either contribute to or represent the primary disruption associated with the development of many lipid-related diseases [7]. In that regard, our group has previously investigated the metabolic aspect of OA by studying the involvement of adipokines and lipid-related genes in its pathogenesis. Osteoarthritic chondrocytes were found to internalize lipids and exhibit reduced expression of genes regulating reverse cholesterol transport, such as ATP-binding cassette transporter A1 (*ABCA1*), apolipoprotein A1 (*ApoA1*), or their transcriptional regulators liver X receptors (*LXRα* and *LXRβ*), resulting in advanced cell toxicity due to accumulation of cholesterol [8-10].

Under normal conditions, biosynthesis of cholesterol is directly regulated by the cholesterol levels present. In OA, we have recently shown that sterol regulatory element-binding protein 2 (SREBP-2), a transcription factor that activates genes of cholesterol metabolism and biosynthesis, such as 3-hydroxy-3-methylglutaryl-coenzyme A reductase, was significantly elevated. We also provided evidence for its induction by transforming growth factor-β1 (TGF-β1) through the phosphoinositide 3-kinase (PI3K)/Akt pathway, a molecular mechanism responsible for the increase in cholesterol synthesis observed in OA [11].

All of the above information suggests that both processes—cholesterol synthesis and cholesterol efflux—are deregulated and contribute to OA pathogenesis. However, so far, the underlying mechanisms for their interaction remain unknown.

In an attempt to open novel avenues in therapeutic strategies for OA, a lot of studies have focused on microRNAs (miRNAs), a rapidly evolving research field. miRNAs are small (20 to 24 nucleotides long), noncoding RNAs that control gene expression at the posttranscriptional level. The mature miRNAs bind to specific, fully or partially complementary sequences in the 3′ untranslated regions (3′-UTRs) of mRNA targets and promote their degradation or prohibit their translation into functional proteins [12-14]. In rare cases, the interaction of miRNAs with a target mRNA takes place at the 5′-UTR or at protein-coding regions [14]. Most miRNA target sites have perfect pairing to the region near the miRNA 5′ end (seed region) or to the region near the miRNA 3′ end (3′ compensatory pairing). Interestingly, “centered pairing,” a unique class of miRNA target sites, has been identified, in which the interaction between miRNA and mRNA takes place in the central region of the miRNA [15].

Recent studies have identified the expression profiles of miRNAs that regulate matrix genes or signaling pathways pertinent to OA [16-20], with specific miRNAs related to both cartilage and adipose tissue biology [20,21]. miRNA-33a (miR-33a) is highly conserved in many animal species and is one of the master regulators of cholesterol and fatty acid metabolism [22], and it is located within intron 16 of the human *SREBP2* gene. It regulates the expression of genes involved in cholesterol export and high-density lipoprotein (HDL) biogenesis (*ABCA1*, *ABCG1* and *NPC1*) [23-25], fatty acid oxidation (*CPT1A*, *CROT*, *HADHB* and *AMPKa*), bile secretion (*ABCB11* and *ATP8B1*) and insulin signaling (*IRS2* and *SIRT6*) [26-28]. More specifically, miR-33a has been demonstrated to have an essential effect on regulating cholesterol metabolism in cooperation with its host gene, *SREBP2*, by strongly repressing the levels of ABCA1 and thus dampening cellular cholesterol efflux to ApoA1 in human and murine macrophages and hepatic cells. Contrarily, inhibition of endogenous miR-33a increases plasma HDL levels through positive regulation of ABCA1 expression [23,25,29-31], serving as useful tool for treating dyslipidemia, cardiovascular disorders and related metabolic diseases.

However, the role of miR-33a in regulating cholesterol homeostasis in OA has not been investigated yet. In this study, we demonstrate that miR-33a expression levels are significantly increased in OA chondrocytes compared with normal chondrocytes, being induced by TGF-β1, and that this miRNA regulates cholesterol synthesis and cholesterol efflux-related genes in OA chondrocytes.

## Methods

### Bioinformatics approaches

The online miRNA databases TargetScan 6.2 [32], miRanda [33] and miRDB [34] were used to search miR-33a target genes.

### Osteoarthritic and normal articular cartilage samples

Cartilage tissues were aseptically obtained from patients with primary OA undergoing total knee replacement surgery at the Department of Orthopaedics of University Hospital of Larissa. A total of 14 patients were included in this study (11 women and 3 men; mean age: 69.9 ± 7.3 years). Radiographs were obtained before surgery and graded using the Kellgren-Lawrence system according to the following criteria: grade 1 (doubtful narrowing of joint space and possible osteophytes), grade 2 (definite osteophytes and possible narrowing of joint space), grade 3 (moderate multiple osteophytes, definite narrowing of joint space and some sclerosis and possible deformity of bone ends) and grade 4 (large osteophytes, marked narrowing of joint space, severe sclerosis and definite deformity of bone ends). All patients had a Kellgren-Lawrence grade ≥2. The assessment of the radiographs by two independent expert observers was blinded. Patients with rheumatoid arthritis and other autoimmune diseases, as well as chondrodysplasias, infection-induced OA and posttraumatic OA, were not included in the study. Normal articular cartilage was obtained from nine individuals (five women and four men; mean age: 66 ± 4.4 years) undergoing fracture repair surgery who had no history of joint disease and did not show clinical manifestations compatible with OA when this was specifically explored by radiographs. Both patients’ and healthy individuals’ cartilage samples were obtained upon their providing written informed consent. The method of obtaining consent was approved by the Institutional Review Board of the University Hospital of Larissa. The study protocol conformed to the ethical guidelines of the 1975 Declaration of Helsinki as reflected in *a priori* approval by the local ethics committee of the University Hospital of Larissa.

### Primary cultures of normal and osteoarthritic articular chondrocytes

Cartilage samples were cut into small pieces with a scalpel and digested at 37°C with 1 mg/ml pronase (Roche Applied Science, Mannheim, Germany) for 30 minutes, and then each sample was centrifuged and the pellet was incubated with 1 mg/ml collagenase P (Roche Applied Science) for 3 hours at 37°C. Chondrocytes were counted and checked for viability by trypan blue staining. More than 95% of the cells were viable after isolation. The isolated chondrocytes were seeded in 25-cm^2^ culture flasks and incubated with Dulbecco’s modified Eagle’s medium/Ham’s F-12 (DMEM/F-12) (GIBCO; Life Technologies, Paisley, UK) plus 5% fetal bovine serum (GIBCO; Life Technologies) and 100 U/ml penicillin-streptomycin (HyClone Laboratories, Logan, UT, USA) at 37°C in an atmosphere of 5% CO_2_ until reaching confluence.

### RNA extraction

Total cellular RNA containing miRNA was extracted from cultured chondrocytes using TRIzol reagent (Invitrogen/Life Technologies). RNA was further purified using an RNeasy Mini Kit (Qiagen, Hilden, Germany). Preservation of 28S and 18S rRNA species was used to assess RNA integrity. All the samples included in the study had prominent 28S and 18S rRNA components. The yield was quantified spectrophotometrically.

### Reverse transcription

For RT-PCR experiments, 1 μg of RNA from each sample was used. Reverse transcription was conducted using the SuperScript III Reverse Transcriptase kit (Invitrogen/Life Technologies) according to the protocol provided by the manufacturer. Osteoarthritic and normal chondrocyte samples were reverse-transcribed using random primers (Invitrogen/Life Technologies), miR-33a stem-loop RT primer (5 pmol in 20-μl reaction volume) or U6 small nuclear RNA (RNU6B) stem-loop RT primer (5 pmol in 20-μl reaction volume) to generate the cDNA according to the method described by Chen *et al*. [35]. Stem-loop primers carried a 3′ overhang of six or seven nucleotides complementary to the 3′ portion of the respective mature miRNA sequence.

### Quantitative RT-PCR

Expression of *ABCA1*, *ApoA1*, *LXRα*, *LXRβ*, *SREBP-2*, matrix metalloproteinase (*MMP*)-13, *Smad7*, glyceraldehyde 3-phosphate dehydrogenase (*GAPDH*), mature miR-33a and RNU6B was determined by real-time PCR (ABI 7300; Applied Biosystems, Foster City, CA, USA). Reactions were done in triplicate using 2 μl of cDNA per reaction. The reactions for miRNA or mRNA were performed in a 10-μl final volume containing 2 μl of cDNA (conducted with stem-loop primer (dilution 1:100) or with random primers (dilution 1:5)), 5 μl of Power SYBR Green PCR Master Mix (Applied Biosystems), 0,3 μl of each primer (forward and reverse) and 2.4 μl of nuclease-free water. All primers used are shown in Table 1. To quantify the relative expression of each miRNA or gene, threshold cycle (*C*_t_) values were normalized against the endogenous reference (Δ*C*_t_ = *C*_t_ (miR-33a) − *C*_t_ (U6)) or Δ*C*_t_ = *C*_t_ (target) − *C*_t_ (GAPDH)) and were compared with a calibrator using the 2^−ΔΔ*C*t^ method (2^−ΔΔ*C*t^ = Δ*C*_t_ (sample) − Δ*C*_t_ (calibrator)).

**Table 1:** **Oligonucleotide primers used in cDNA synthesis for the detection of miR-33a and U6 (stem-loop primers) and for real-time quantitative PCR assay** ^**a**^

	**RT-qPCR stem-loop primer**	
miR-33a	5′-TGGATATCCACACCAGGGTCCGAGGTATTCGGTGTGGATATCCATGCAATG	
U6	5′-CACGGAAGCCCTCACACCGTGTCGTTC	
**Gene**	**Forward primer sequence**	**Reverse primer sequence**
*miR-33a*	CGCGCGTGCATTGTAGTTG	CACCAGGGTCCGAGGT
*U6*	GCTTCGGCAGCACATATACTAAAAT	CTCACACCGTGTCGTTCCA
*SREBP2*	AAGTCTGGCGTTCTGAGGAA	AGGTCCACCTCATTGTCCAC
*ABCA1*	GGAGGCAATGGCACTGAGGAA	CCTGCCTTGTGGCTGGAGTGT
*ApoA1*	ATGAAAGCTGCGGTGCTGACC	CACCTTCTGGCGGTAGAGCTCC
*LXRα*	CCGCCTGAAGAAACTGAA	CGAAGCCGGTCAGAAAAG
*LXRβ*	CGCTACAACCACGAGACAGA	GTGGAAGTCGTCCTTGCTGT
*MMP-13*	TGGCATTGCTGACATCATGA	GCCAGAGGGCCCATCAA
*Smad7*	TCCAGATACCCGATGGATTTTC	GATTTTGCTCCGCACCTTCT
*GAPDH*	GAGTCAACGGATTTGGTCGT	GACAAGCTTCCCGTTCTCAG

^a^ABCA1, ATP-binding cassette transporter A1; ApoA1, Apolipoprotein A1; GAPDH, Glyceraldehyde 3-phosphate dehydrogenase; LXR, Liver X receptor; miR-33a, MicroRNA-33a mature; MMP-13, Matrix metalloproteinase-13; SREBP2, Sterol regulatory element-binding protein 2.

### Protein extraction and Western blot analysis

Chondrocytes were lysed using lysis buffer containing 30 mM Tris (pH 7.5), 150 mM NaCl, 10% glycerol, 1% Nonidet P-40 and a cocktail of protease and phosphatase inhibitors (Roche Applied Science). Protein concentration was quantified using the Bradford protein assay (Bio-Rad Laboratories, Hercules, CA, USA) with bovine serum albumin as standard. Cell lysates from chondrocytes were electrophoresed and separated on 10% acrylamide gels and transferred to polyvinylidene fluoride membranes (EMD Millipore, Billerica, MA, USA) that were probed with anti-total Akt (Santa Cruz Biotechnology Europe, Heidelberg, Germany) and anti-p-Akt (Abcam, Cambridge, UK). Signals were detected using suitable immunoglobulin G conjugated with horseradish peroxidase. Western blot bands were quantified using ImageJ software (National Institutes of Health, Bethesda, MD, USA).

### Treatment with TGF-β1

Primary cultured human chondrocytes were seeded onto six-well plates at a density of 3 × 10^5^ cells/well. Three days postseeding, normal chondrocytes were serum-starved overnight and then cultured in serum-free DMEM/F-12 in the presence or absence of 10 ng/ml TGF-β1 (Sigma-Aldrich, St Louis, MO, USA) for 0.5 hours, 2 hours, 6 hours, 24 hours and 48 hours. Each experiment was conducted in triplicate, and the results from three wells were averaged and considered as n = 1. RNA was extracted, and real-time PCR analysis was performed (6 hours, 24 hours, 48 hours). Total and phospho-proteins were extracted, and Western blot analysis was performed (0.5 hours, 2 hours, 24 hours).

### TGF-β1 treatment in normal chondrocytes with subsequent use of miR-33a inhibitor

Cells were seeded onto six-well plates at a density of 3 × 10^5^cells/well. Three days postseeding, normal chondrocytes were serum-starved overnight and then cultured in serum-free DMEM/F-12 in the presence or absence of 10 ng/ml TGF-β1 (Sigma-Aldrich) for 6 hours. After 6-hour culture of normal chondrocytes with TGF-β1, the culture medium was changed and treated with 50 nM anti-miR-33a or negative control for 24 hours. Each experiment was conducted in duplicate, and the results from two wells were averaged and considered as n = 1. RNA was extracted, and real-time PCR analysis was performed.

### Transient transfection of microRNA mimic and inhibitor

miRNA mimic (miR-33a), antagomir (anti-miR-33a) or negative control oligonucleotides were obtained from Ambion/Life Technologies. Primary cultured normal human chondrocytes were transfected with 30 nM miR-33a mimic using Lipofectamine 2000 reagent (Invitrogen/Life Technologies) for 6 hours, 24 hours and 48 hours. miR-33a inhibitor (50 nM) was transfected into human osteoarthritic chondrocytes for 24 and 48 hours. Each experiment was conducted in triplicate, and the results from three wells were averaged and considered as n = 1. RNA was extracted, and real-time PCR was performed as previously described.

### Transient transfection of microRNA inhibitor with subsequent TGF-β1 treatment and transient transfection of miR-33a mimic plus TGF-β1 in human chondrocytes

Cells were seeded onto six-well plates at a density of 3 × 10^5^cells/well. Three days postseeding, OA chondrocytes were serum-starved overnight and then cultured in serum-free DMEM/F-12 in the presence or absence of 50 nM anti-miR-33a for 24 hours. The culture medium was changed and treated with 10 ng/ml TGF-β1 (Sigma-Aldrich) for 2 hours. Normal chondrocytes were serum-starved overnight and then cultured in serum-free DMEM/F-12 in the presence or absence of 30 nM miR-33a and 10 ng/ml TGF-β1 (Sigma-Aldrich) or 10 ng/ml TGF-β1 alone for 24 hours. Each experiment was conducted in duplicate, and the results from two wells were averaged and considered as n = 1. Total and phospho-proteins were extracted, and Western blot analysis was performed.

### Statistical analysis

All statistical analysis was performed using SPSS Statistics 20 software (IBM, Armonk, NY, USA). Gene expression data were analyzed using Student’s *t*-test and a confidence level of 95%. Numerical data were expressed as mean ± standard error mean (SEM). *P* < 0.05 was considered statistically significant.

## Results

### miR-33a expression is elevated in OA chondrocytes

**Figure 1:**
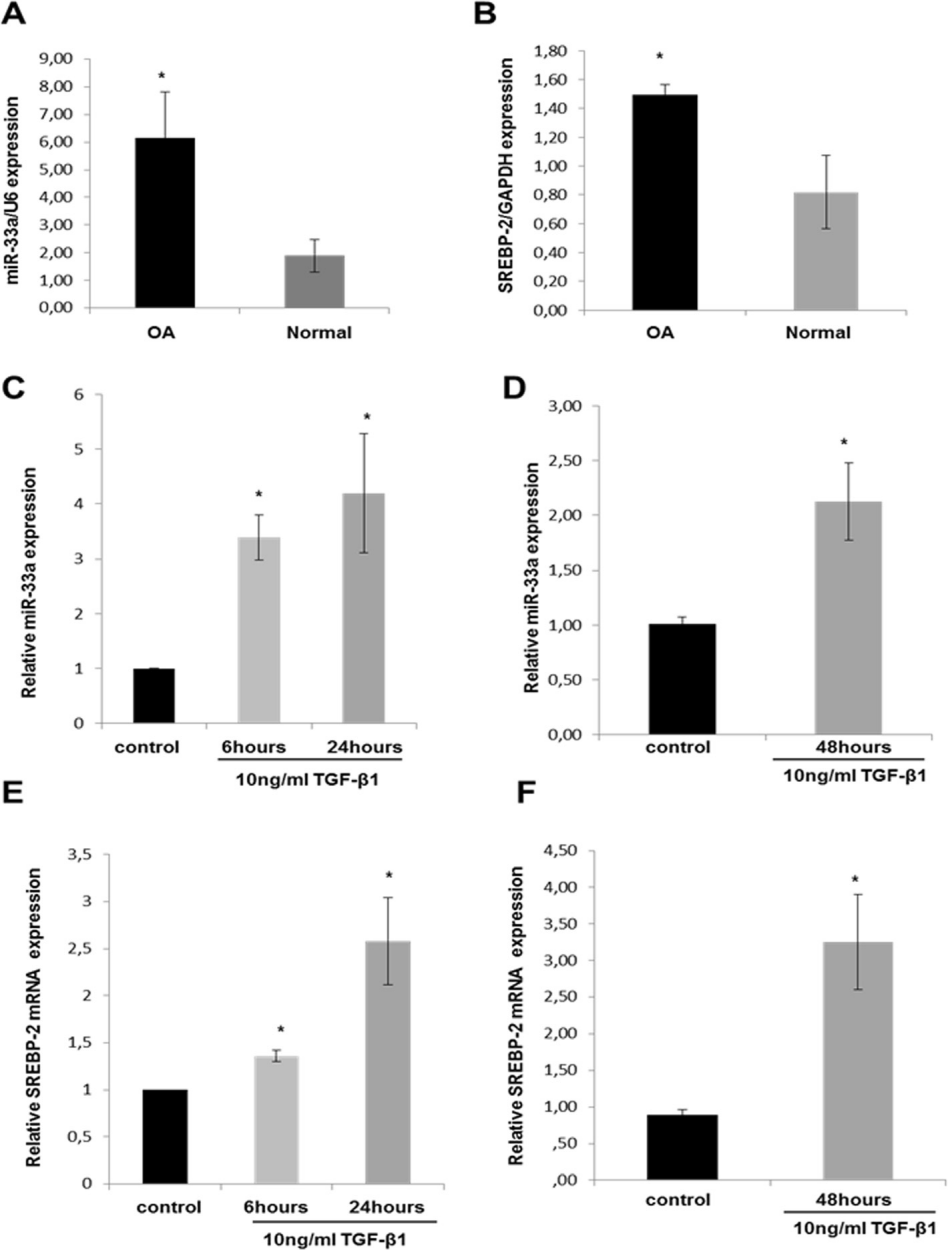
**MicroRNA-33a is elevated in osteoarthritic chondrocytes and is induced by transforming growth factor-β1. (A)** Relative expression of microRNA (miR)-33a in normal and osteoarthritic (OA) chondrocytes (n = 9 for normal chondrocytes from 9 different donors, n = 14 for OA chondrocytes from 14 different donors). U6 was used for normalization of the real-time PCR data. The data are expressed as mean and standard error of the mean (SEM) of three independent experiments, each of which was run in duplicate. *P < 0.05 as measured using an unpaired Student’s *t*-test. **(B)** Relative expression of sterol regulatory element-binding protein 2 (*SREBP-2*) in normal and OA chondrocytes (n = 5 for normal chondrocytes from 5 different donors, n = 10 for OA chondrocytes from 10 different donors). Glyceraldehyde 3-phosphate dehydrogenase (*GAPDH*) was used for normalization of the real-time PCR data. The data are expressed as mean and SEM of three independent experiments, each of which was run in duplicate. *P < 0.05 as measured using an unpaired Student’s *t*-test. **(C)** and **(D)** miR-33a expression levels in cultured normal chondrocytes (n = 4 from 4 different donors) following treatment with 10 ng/ml transforming growth factor (TGF)-β1 for 6 hours, 24 hours **(C)** and 48 hours **(D)**. U6 was used for normalization of the real-time PCR data. The data are expressed as mean and SEM of two independent experiments, each of which was run in triplicate. *P < 0.05 compared with control. **(E)** and **(F)** *SREBP-2* expression levels in cultured normal chondrocytes (n = 5 from 5 different donors) following treatment with 10 ng/ml TGF-β1 for 6 hours, 24 hours **(E)** and 48 hours **(F)**. *GAPDH* was used for normalization of the real-time PCR data. The data are expressed as mean and SEM of two independent experiments, each of which was run in triplicate. *P < 0.05 compared with control.

There is evidence that intronic miRNAs are coordinately expressed and processed with the precursor mRNA in which they reside [36]. Taking into consideration the fact that miR-33a is located within intron 16 of the human *SREBP2* gene and that *SREBP2* expression is upregulated in OA [11], we wanted to test whether miR-33a and its host gene *SREBP2* are coexpressed in human chondrocytes. We evaluated their expression levels by quantitative RT-PCR and found that they were both significantly elevated in OA chondrocytes compared with normal chondrocytes (*P* < 0.05) (Figure 1A,B).

### TGF-β1 induces miR-33a expression in human chondrocytes

We have recently shown that SREBP-2 is activated by TGF-β1 in human chondrocytes through the integrin alpha-V/PI3K/Akt pathway [11]. We next proceeded to investigate whether miR-33a expression is also induced by this growth factor. Normal chondrocytes were treated with 10 ng/ml TGF-β1 for 6 hours, 24 hours and 48 hours, and we found that both miR-33a and *SREBP-2* expression levels were significantly upregulated in chondrocytes treated with TGF-β1 compared with untreated cells (*P* < 0.05) (Figure 1C–F).

### Regulation of SREBP-2 expression by anti-miR-33a in TGF-β1-induced chondrocytes

**Figure 2:**
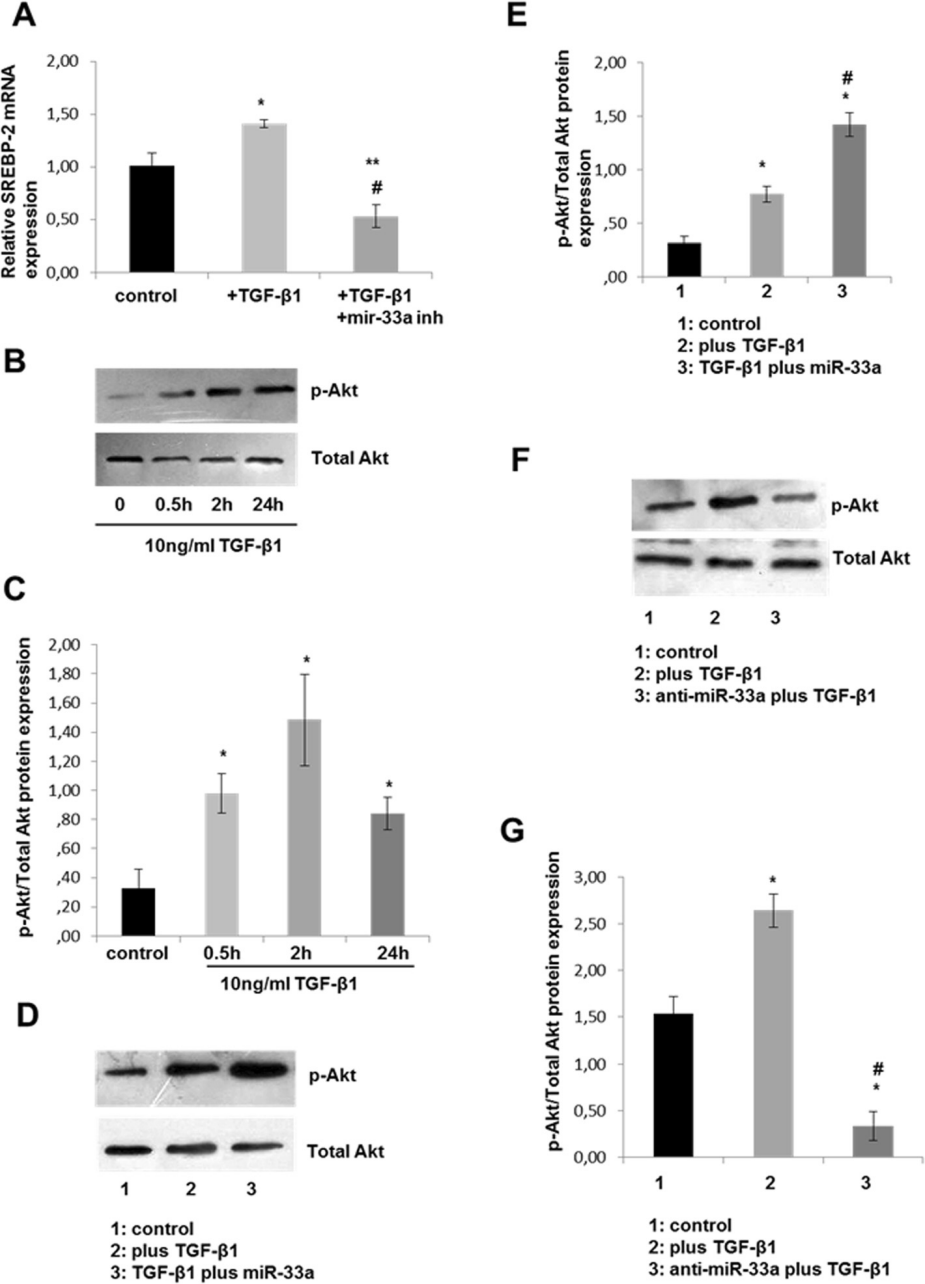
**Effect of microRNA-33a on transforming growth factor-β1-mediated phosphoinositide 3-kinase/Akt signaling pathway in human chondrocytes. (A)** Sterol regulatory element-binding protein 2 (*SREBP-2*) mRNA expression in human normal chondrocytes (n = 3 from 3 different donors) after treatment with 10 ng/ml transforming growth factor (TGF)-β1 with or without microRNA (miR)-33a inhibitor. Glyceraldehyde 3-phosphate dehydrogenase (*GAPDH*) was used for normalization of the real-time PCR data. The data are expressed as mean and SEM from triplicates of one representative of three experiments. *P < 0.05 versus negative control, **P < 0.01, #TGF-β1 with miR-33a inhibitor versus TGF-β1. **(B)** Western blot showing phosphorylated (p-Akt) and total Akt protein expression levels in normal chondrocytes treated with 10 ng/ml TGF-β1 for 0.5, 2 and 24 hours. **(C)** Diagram showing p-Akt protein expression normalized to total Akt using ImageJ software. The result shown represents the mean from three different blots. *P < 0.05 compared with control. **(D)** Western blot showing p-Akt and total Akt protein expression levels in normal chondrocytes treated with TGF-β1, TGF-β1 plus miR-33a or negative control. **(E)** Diagram showing p-Akt protein expression normalized to total Akt using ImageJ software. The result shown represents the mean from three different blots. *P < 0.05 compared with control. #TGF-β1 with miR-33a versus TGF-β1. **(F)** Western blot showing p-Akt and total Akt protein expression levels in osteoarthritis chondrocytes treated with TGF-β1 and miRNA inhibitor (anti-miR-33a), followed by treatment of TGF-β1 or negative control. **(G)** Diagram showing p-Akt protein expression normalized to total Akt using ImageJ software. The result shown represents the mean from three different blots. *P < 0.05 compared with control. #TGF-β1 with miR-33a inhibitor versus TGF-β1.

Because TGF-β1 was found to upregulate *SREBP-2* and miR-33a, normal chondrocytes were treated with 10 ng/ml TGF-β1 for 6 hours. After that period of time, chondrocytes were transfected with 50 nM miR-33a inhibitor (anti-miR-33a) for 24 hours. Our results showed that *SREBP-2* expression levels were reduced in chondrocytes treated with 10 ng/ml TGF-β1 together with 50 nM anti-miR-33a compared with TGF-β1 treatment (*P* < 0.001) (Figure 2A).

### PI3K/Akt pathway is directly associated with TGF-β receptor

To show whether the PI3K/Akt pathway is directly or indirectly associated with the TGF-β receptor, we treated normal chondrocytes with 10 ng/ml TGF-β1 over different periods of time (from 0.5 to 24 hours) and found a rapid induction of Akt phosphorylation, suggesting direct involvement of PI3K in TGF-β-receptor induced intracellular signaling (Figure 2B,C).

### Regulation of TGF-β1-induced Akt phosphorylation by miR-33a

Because miR-33a expression was significantly increased by TGF-β1 stimulation in human chondrocytes and its inhibition reduced *SREBP-2* expression levels, we were prompted to investigate miR-33a’s role in the PI3K/Akt pathway. Transfection of normal chondrocytes with 10 ng/ml TGF-β1 plus 30 nM miR-33a for 24 hours resulted in significant increased Akt phosphorylation compared with TGF-β1 treatment alone (Figure 2D,E). Inhibition of miR-33a in human chondrocytes inhibited TGF-β1-induced Akt phosphorylation. Total Akt expression was not changed by transfection of miR-33a or miR-33a inhibitor (Figure 2F,G).

### miR-33a modulates TGF-β1 induced PI3K/Akt signaling pathway by targeting Smad7

**Figure 3:**
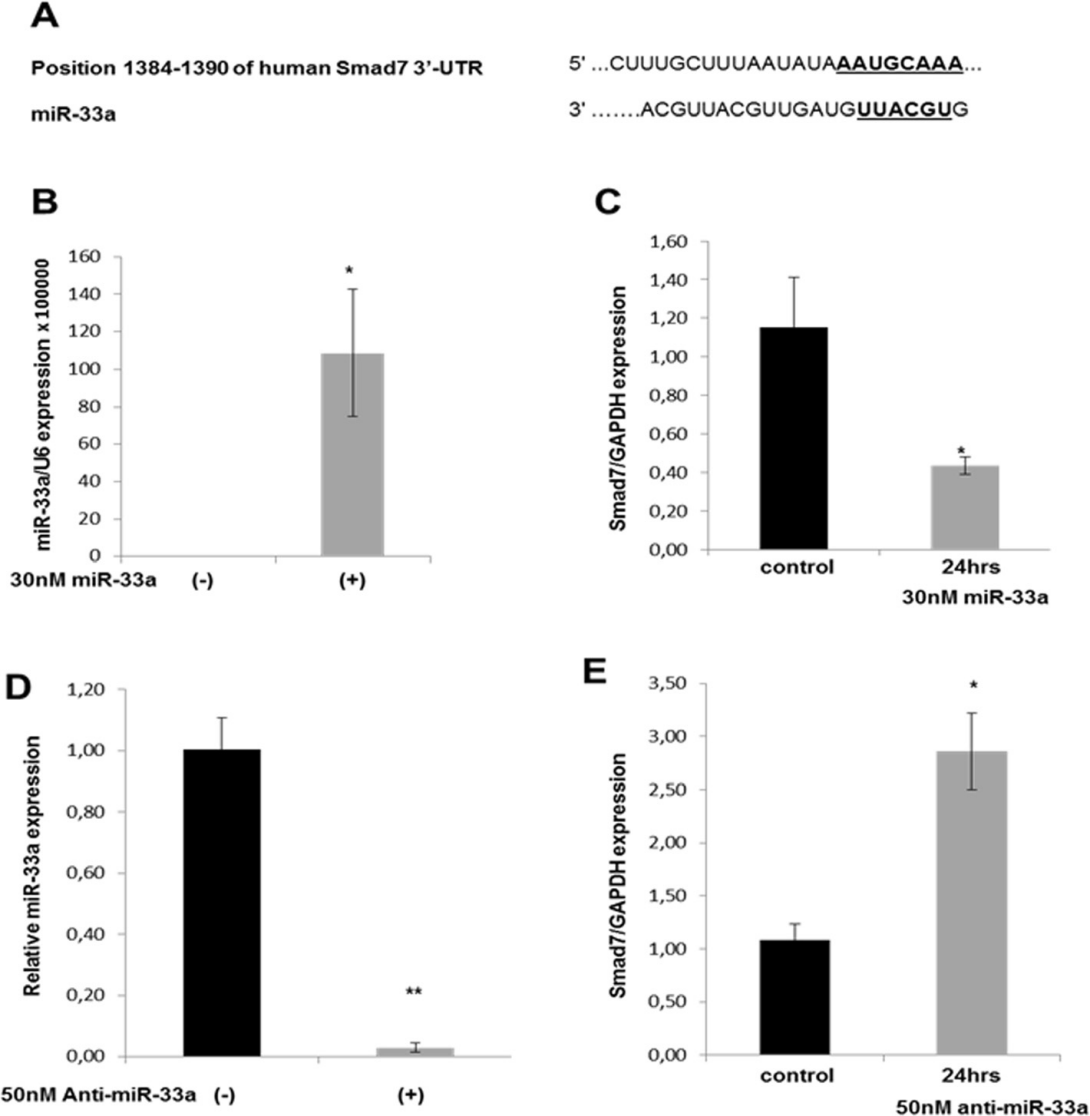
**ΜicroRNA-33a affects Smad7 expression in human chondrocytes. (A)** TargetScan 6.2 and miRanda identified one conserved sequence in the 3′ untranslated region of human *Smad7* mRNA that was completely complementary to microRNA (miR)-33a. **(B)** Relative expression levels of miR-33a in normal human chondrocytes (n = 3 from 3 different donors) transfected with miR-33a mimic or negative control. U6 was used for normalization of the real-time PCR data. The data are expressed as mean and standard error of the mean (SEM) of three independent experiments, each of which was run in duplicate. *P < 0.05 versus negative control. **(C)** Relative expression levels of *Smad7* mRNA 24 hours after transfection of 30 nM miR-33a or negative control in normal human chondrocytes (n = 6 from 6 different donors). Glyceraldehyde 3-phosphate dehydrogenase (*GAPDH*) was used for normalization of the real-time PCR data. The data are expressed as mean and SEM of three independent experiments, each of which was run in triplicate. *P < 0.05 versus negative control. **(D)** Relative expression levels of miR-33a in human osteoarthritis (OA) chondrocytes transfected with miR-33a inhibitor or negative control (n = 3 from 3 different donors). U6 was used for normalization of the real-time PCR data. The data are expressed as mean and SEM of three independent experiments, each of which was run in duplicate. *P < 0.05 versus negative control. **(E)** Relative expression levels of *Smad7* mRNA 24 hours after transfection of 50 nM anti-miR-33a or negative control in human OA chondrocytes (n = 4 from 4 different donors). *GAPDH* was used for normalization of the real-time PCR data. The data are expressed as mean and SEM of three independent experiments, each of which was run in triplicate. *P < 0.05 versus negative control.

Taking into consideration the fact that the degree of activation of the TGF-β signaling pathways is subject to regulation by a large number of intracellular and extracellular agonists and antagonists, including Smad7 and Smurf, we performed computational analysis of the 3′ UTR of *Smad7* mRNA, a negative regulator of TGF-β signaling. The TargetScan 6.2 and miRanda prediction tools showed that *Smad7* is a target gene of miR-33a (Figure 3A).

To verify this prediction, we transfected normal chondrocytes with 30 nM miR-33a mimic for 24 hours. Treatment of normal chondrocytes with miR-33a mimic resulted in significant upregulation of miR-33a expression levels compared with negative control (*P* < 0.05) (Figure 3B). We observed a significant reduction in *Smad7* mRNA expression compared with untreated cells (*P* < 0.05) (Figure 3C). Treatment of OA chondrocytes with 50 nM miR-33a inhibitor resulted in significant suppression of miR-33a (Figure 3D) (*P* < 0.05) and in significantly increased *Smad7* mRNA expression levels compared with negative control (*P* < 0.05) (Figure 3E).

### Computational prediction of miR-33a lipid-related target genes

**Figure 4:**
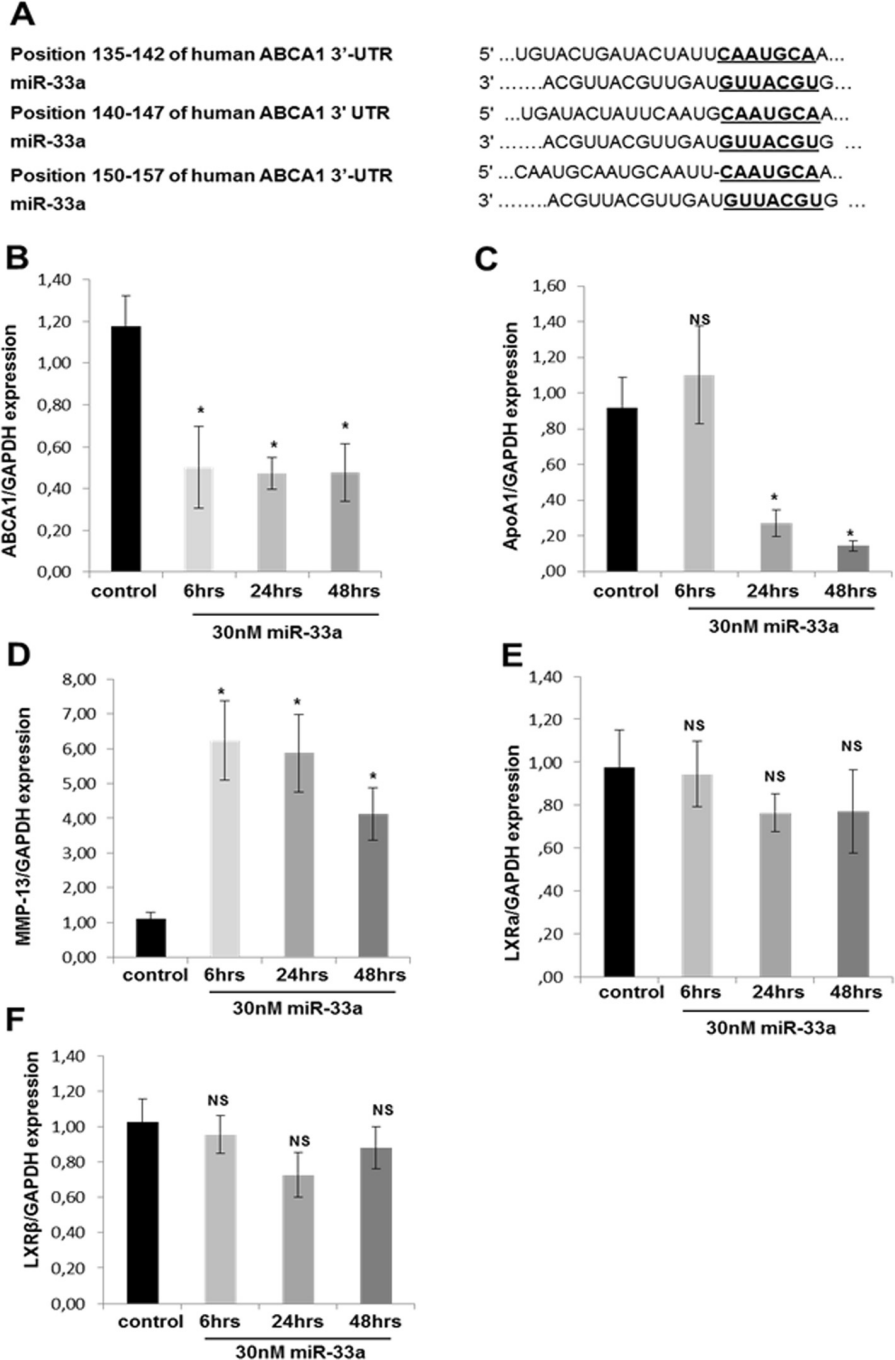
**ΜicroRNA-33a affects ATP-binding cassette transporter A1 and apolipoprotein A1 expression in human chondrocytes. (A)** TargetScan 6.2, miRanda and miRDB identified three conserved sequences in the 3′ untranslated region of human ATP-binding cassette transporter A1 (*ABCA1*) mRNA that were completely complementary to microRNA (miR)-33a. **(B)** and **(C)** Relative expression levels of ABCA1 **(B)** and apolipoprotein A1 (*ApoA1*) **(C)** mRNA at 6, 24 and 48 hours after transfection of 30 nM miR-33a or negative control in normal human chondrocytes (n = 6 from 6 different donors). Glyceraldehyde 3-phosphate dehydrogenase (*GAPDH*) was used for normalization of the real-time PCR data. The data are expressed as mean and standard error of the mean (SEM) of two independent experiments, each of which was run in triplicate. *P < 0.05 versus negative control. **(D)** Relative expression levels of matrix metalloproteinase (*MMP*)-13 mRNA at 6, 24 and 48 hours after transfection of 30 nM miR-33a or negative control in normal human chondrocytes (n = 4 from 4 different donors). *GAPDH* was used for normalization of the real-time PCR data. The data are expressed as mean and SEM of two independent experiments, each of which was run in triplicate. *P < 0.05 versus negative control. **(E)** and **(F)** Relative expression levels of liver X receptor α (*LXRα*) **(E)** and *LXRβ* **(F)** mRNA at 6, 24 and 48 hours after transfection of 30 nM miR-33a or negative control in normal human chondrocytes (n = 4 from 4 different donors). *GAPDH* was used for normalization of the real-time PCR data. The data are expressed as mean and SEM of two independent experiments, each of which was run in triplicate. NS, Not significant.

To investigate the role of miR-33a in regulating lipid-related genes, we used bioinformatics prediction tools, which identified three conserved sequences in the 3′-UTR of human *ABCA1* mRNA that were completely complementary to miR-33a (Figure 4A). None of the other genes examined (*ApoA1*, *SREBP2*, *LXRα*, *LXRβ*) had a fully or partially complementary sequence to miR-33a (data not shown).

### miR-33a targets to ATP-binding cassette transporter A1 and suppresses its expression in human chondrocytes

To examine the role of miR-33a in the expression of genes regulating reverse cholesterol transport (*ABCA1*, *ApoA1*, *LXRα* and *LXRβ*), normal chondrocytes were transfected with 30 nM miR-33a mimic for 6 , 24 and 48 hours. miR-33a treatment significantly suppressed *ABCA1* mRNA expression levels at 6, 24 and 48 hours (*P* < 0.05) (Figure 4B), as well as *ApoA1* mRNA expression levels at 24 and 48 hours (*P* < 0.05) (Figure 4C), which was accompanied by elevated levels of *MMP-13* (*P* < 0.05) (Figure 4D). Transfection of miR-33a mimic had no effect on the expression levels of *LXRα* and *LXRβ* in human chondrocytes (*P* > 0.05) (Figure 4E,F).

### Anti-miR-33a induces ATP-binding cassette transporter A1 expression in human chondrocytes

**Figure 5:**
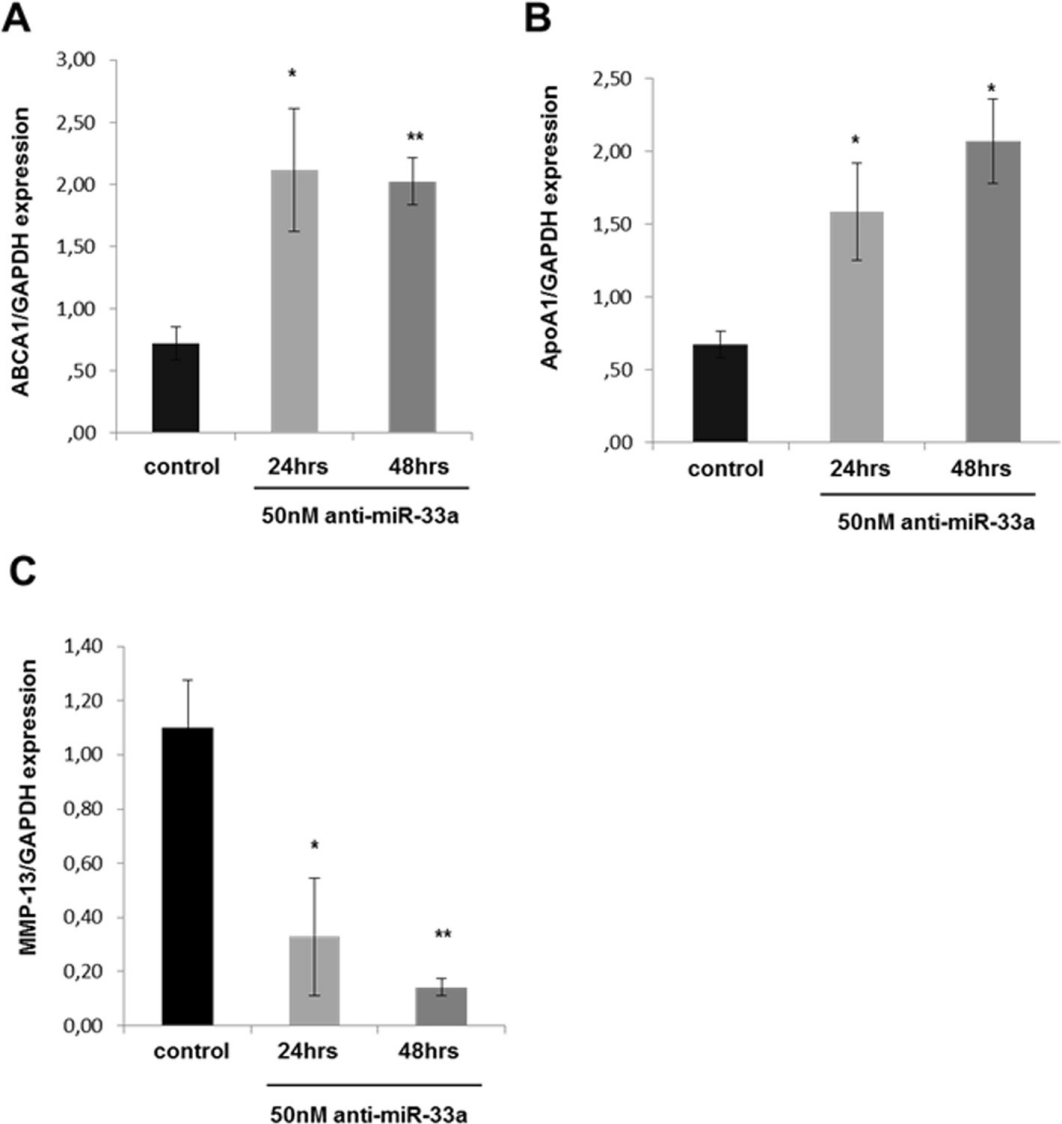
**Anti-microRNA-33a affects ATP-binding cassette transporter A1 and apolipoprotein A1 expression in human chondrocytes. (A)** Relative expression levels of ATP-binding cassette transporter A1 (*ABCA1*) mRNA 24 and 48 hours after transfection of 50 nM anti-microRNA (miR)-33a or negative control in human osteoarthritis (OA) chondrocytes (n = 5 from 5 different donors). Glyceraldehyde 3-phosphate dehydrogenase (*GAPDH*) was used for normalization of the real-time PCR data. The data are expressed as mean and standard error of the mean (SEM) of two independent experiments, each of which was run in triplicate. *P < 0.05 versus negative control. **(B)** Relative expression levels of apolipoprotein A1 (*ApoA1*) mRNA 24 and 48 hours after transfection of 50 nM anti-miR-33a or negative control in human OA chondrocytes (n = 5 from 5 different donors). *GAPDH* was used for normalization of the real-time PCR data. The data are expressed as mean and SEM of two independent experiments, each of which was run in triplicate. *P < 0.05 versus negative control. **(C)** Relative expression levels of matrix metalloproteinase (*MMP*)-13 mRNA 24 and 48 hours after transfection of 50 nM anti-miR-33a or negative control in human OA chondrocytes (n = 5 from 5 different donors). *GAPDH* was used for normalization of the real-time PCR data. The data are expressed as mean and SEM of two independent experiments, each of which was run in triplicate. *P < 0.05 versus negative control.

To confirm that miR-33a regulates cholesterol efflux-related genes in a manner that can be reversed by its inhibitor, OA chondrocytes were treated with 50 nM anti-miR-33a for 24 and 48 hours, and expression levels of *ABCA1*, *ApoA1* and *MMP-13* were evaluated. Treatment of OA chondrocytes with anti-miR-33a resulted in significantly upregulated *ABCA1* and *ApoA1* mRNA expression levels (*P* < 0.05) (Figure 5A,B), whereas *MMP-13* mRNA expression levels were reduced (*P* < 0.05) (Figure 5C).

## Discussion

A growing body of evidence suggests that the relationship between MetS and OA is not one of cause and effect, but rather is indicative of an underlying common set of factors, highlighting OA as a metabolic disorder [3-6,10,37-39]. miRNAs have key roles in modulating and maintaining normal physiological conditions, with their signaling emerging as of great interest as a potential tool for diagnosis of and therapy for the progression of a number of diseases, such as OA.

miR-33a plays a crucial role in controlling cholesterol homeostasis in metabolism-related disorders [26], but so far no association with OA has been found. It is embedded within intron 16 of *SREBP2*, a lipid metabolism-related gene, which has been identified by our group to be involved in OA pathogenesis [11].

In the present study, we investigated the role of miR-33a in cholesterol synthesis and cholesterol efflux-related genes. We found that miR-33a expression levels were significantly elevated in osteoarthritic chondrocytes compared with normal chondrocytes, in accordance to *SREBP-2* upregulation. In line with previous findings in human and mouse tissues, such as HEK293 cells, Hep3B human hepatoma cells and mouse peritoneal macrophages [23,29,30,40], our results also suggest that miR-33a is coexpressed with its host gene in human chondrocytes.

As we previously reported that SREBP-2 can be induced by TGF-β1 in chondrocytes, we proceeded to investigate whether TGF-β1 activates miR-33a as well. We found that miR-33a expression was increased in a time-dependent manner after TGF-β1 activation, suggesting that the expression levels of miR-33a and *SREBP-2* are coregulated by TGF-β1 at the transcriptional level. This finding is in accordance with recent studies which showed that the expression of both miR-33a and SREBP-2 was increased in a dose- and time-dependent way after TGF-β1 stimulation in immortalized human hepatic stellate cells. We also showed that normal chondrocytes stimulated with TGF-β1, followed by transient transfection of anti-miR-33a, exhibited reduced expression levels of *SREBP-2*, supporting the strong association between *SREBP-2* and its intronic miRNA, miR-33a [23,29,30].

Furthermore, we have also previously shown that the induction of SREBP-2 by TGF-β1 in human chondrocytes is mediated through the PI3K/Akt pathway [11]. To show whether the PI3K/Akt pathway is directly or indirectly associated with the TGF-β receptor, we treated normal chondrocytes with TGF-β1 and found rapid induction of Akt phosphorylation, supporting the direct involvement of PI3K in TGF-β receptor-induced intracellular signaling [41,42]. This pathway is important in regulating the production of MMPs by chondrocytes [43]. It has also been suggested to be implicated in different pathological conditions, cancer, diabetes, viral infections [44] and, more recently, hepatic fibrosis, and thus it is an important player in the regulation of lipid metabolism [45]. We found that transient transfection of human chondrocytes with miR-33a plus TGF-β1 and enhanced Akt phosphorylation, whereas inhibition of miR-33a resulted in subsequent inhibition of TGF-β1-induced Akt phosphorylation. Our previous result is in accordance with the study by Li *et al*., who reported that inhibition of miR-33a in LX-2 cells inhibited the phosphorylation of Akt after TGF-β1 treatment [45]. The above findings suggest the involvement of miR-33a in the regulation of the TGF-β1/PI3K/Akt/SREBP-2 signaling pathway responsible for increased cholesterol synthesis in OA.

A growing amount of evidence has demonstrated that a large number of intracellular and extracellular activators and inhibitors regulate the degree of activation of the TGF-β signaling pathways. Among them, Smad7, an inhibitory Smad, is a key regulator of TGF-β signaling [42,46-48]. In the present study, we showed that miR-33a targets *Smad7* in human chondrocytes, suggesting that miR-33a possibly regulates TGF-β1/PI3K/Akt signaling through modulating *Smad7* expression. Interestingly, Huang *et al*. [49] recently demonstrated that Smad7 is targeted by miR-33a in LX-2 cells, thus highlighting its inhibition as a possible mechanism responsible for miR-33a profibrogenic effects in hepatic stellate cells. In an attempt to investigate the role of miR-33a in the regulation of reverse cholesterol transport, we investigated its effect in the cholesterol efflux-related genes *ABCA1*, *ApoA1*, *LXRα* and *LXRβ*. Taking into consideration the facts that *ABCA1* and *ApoA1* expression levels were previously shown by our group to be significantly reduced in OA chondrocytes compared with normal chondrocytes [10] and that *ABCA1* is a direct and specific target of miR-33a, as evidenced by previous studies [23-25,28] and by our bioinformatics analysis, we investigated the effect of this miRNA on genes regulating cholesterol efflux in chondrocytes. We found that treatment of normal chondrocytes with miR-33a resulted in significant reduction of *ABCA1* and *ApoA1* mRNA expression levels, accompanied by increased levels of *MMP-13*. As evidenced by bioinformatics analysis, miR-33a is fully complementary to the 3′-UTR of *ABCA1*, directly resulting in degradation of *ABCA1*’s mRNA transcripts. As *ApoA1* is not a target gene of miR-33a, the reduced expression we observed after miR-33a treatment in normal chondrocytes could be considered an indirect effect caused by the inhibition of *ABCA1*. Interestingly, miR-33a had no effect on *LXRα* and *LXRβ* expression levels, as we showed by bioinformatics analysis that there are no binding sites on the *LXRα* and *LXRβ* gene regions for this miRNA. On the basis of our results, miR-33a emerges as part of a different regulatory mechanism of genes involved in cholesterol efflux, apart from the traditional ligand-activated transcription factor LXR, providing novel evidence of the contribution of miR-33a to the blockage of reverse cholesterol transport in human chondrocytes.

To confirm that miR-33a regulates cholesterol efflux genes in a way that can be reversed by its inhibitor, we treated OA chondrocytes with anti-miR-33a. Introduction of antisense oligonucleotides directed against miR-33a indeed resulted in strongly increased *ABCA1* and *ApoA1* expression levels, and it also caused reduced expression of *MMP-13*, the most abundantly expressed catabolic gene in OA. The above findings are consistent with the reported regulation of ABCA1 by miR-33a in cell lines such as human HepG2 liver carcinoma cells, IMR-90 normal human fibroblasts and the mouse macrophage cell line J774 [24]. Moreover, in studies in animal models of hypercholesterolemia and atherosclerosis, researchers have reported that miR-33a suppresses the expression of *ABCA1* and lowers HDL levels, whereas inhibition of miR-33 increases *ABCA1* and HDL levels [25,30,31].

## Conclusions

We provide novel evidence for the implication of miR-33a, a *SREBP-2* intronic miRNA, in OA pathogenesis, identifying it as a dual regulator of cholesterol synthesis and cholesterol efflux-related genes in OA chondrocytes, suggesting its potential use as a novel target for the amelioration of the OA phenotype.

## Abbreviations

ABCA1: ATP-binding cassette transporter A1
Anti-miR-33a: MicroRNA-33a antagomir
ApoA1: Apolipoprotein A1
DMEM: Dulbecco’s modified Eagle’s medium
GAPDH: Glyceraldehyde 3-phosphate dehydrogenase
HDL: High-density lipoprotein
LXR: Liver X receptor
miR-33a: MicroRNA-33a
miRNA: MicroRNA
MMP: Matrix metalloproteinase
OA: Osteoarthritis
PI3K: Phosphoinositide 3-kinase
RNU6B: U6 small nuclear RNA
SEM: Standard error of the mean
SREBP-2: Sterol regulatory element-binding protein 2
TGF-β1: Transforming growth factor-β1
UTR: Untranslated region
